# Expanded CURB-65: a new score system predicts severity of community-acquired pneumonia with superior efficiency

**DOI:** 10.1038/srep22911

**Published:** 2016-03-18

**Authors:** Jin-liang Liu, Feng Xu, Hui Zhou, Xue-jie Wu, Ling-xian Shi, Rui-qing Lu, Alessio Farcomeni, Mario Venditti, Ying-li Zhao, Shu-ya Luo, Xiao-jun Dong, Marco Falcone

**Affiliations:** 1 Department of Infectious Diseases, Second Affiliated Hospital, Zhejiang University School of Medicine, Hangzhou, China; 2 Experimental Medical Class 1102, Chu Kochen Honor College, China; 3 Department of Public Health and Infectious Diseases, “Sapienza” University of Rome, Italy

## Abstract

Aim of this study was to develop a new simpler and more effective severity score for community-acquired pneumonia (CAP) patients. A total of 1640 consecutive hospitalized CAP patients in Second Affiliated Hospital of Zhejiang University were included. The effectiveness of different pneumonia severity scores to predict mortality was compared, and the performance of the new score was validated on an external cohort of 1164 patients with pneumonia admitted to a teaching hospital in Italy. Using age ≥ 65 years, LDH > 230 u/L, albumin < 3.5 g/dL, platelet count < 100 × 10^9^/L, confusion, urea > 7 mmol/L, respiratory rate ≥ 30/min, low blood pressure, we assembled a new severity score named as expanded-CURB-65. The 30-day mortality and length of stay were increased along with increased risk score. The AUCs in the prediction of 30-day mortality in the main cohort were 0.826 (95% CI, 0.807–0.844), 0.801 (95% CI, 0.781–0.820), 0.756 (95% CI, 0.735–0.777), 0.793 (95% CI, 0.773–0.813) and 0.759 (95% CI, 0.737–0.779) for the expanded-CURB-65, PSI, CURB-65, SMART-COP and A-DROP, respectively. The performance of this bedside score was confirmed in CAP patients of the validation cohort although calibration was not successful in patients with health care-associated pneumonia (HCAP). The expanded CURB-65 is objective, simpler and more accurate scoring system for evaluation of CAP severity, and the predictive efficiency was better than other score systems.

Community-acquired pneumonia (CAP) is one of the most common infectious diseases needing hospitalization. Inappropriate treatment of outpatient or delay of admission of CAP patients to ICU has been shown to be associated with increased mortality^1^,^2^, and it is important for physicians to identify patients who are experiencing severe pneumonia with probably worst prognosis as early as possible. Moreover, pneumonia occurring in patients living in the community but with a recent exposure to the healthcare system (i.e. patients with recent hospitalization, undergoing hemodialysis, or living in nursing homes or long-term care facilities) has been named healthcare-associated pneumonia (HCAP). Several studies suggest that this category of pneumonia has a higher mortality than CAP^3^,^4^.

Multiple serum biomarkers and several established risk scores have been used to assess the severity of CAP to improve management of CAP patients. Pneumonia Severity Index (PSI) was the first scoring system, which consists of twenty clinical and laboratory parameters and is recommended by the American Thoracic Society (ATS)/Infectious Diseases Society of America (IDSA)^5^. CAP patients can be assigned into 5 risk classes. Patients with class IV–V should be hospitalized for treatment as the prognosis deteriorates along with increasing risk class. Although the PSI exhibits a high discriminatory power for assigning appropriate risk class, it is complicated to calculate and limits clinical application. Later, the British Thoracic Society recommended a system using Confusion, Urea, Respiratory rate, Blood pressure plus age ≥ 65 years (CURB-65) for CAP management^6^. CURB-65 simplifies the scoring system compared with PSI, but at the expense of reducing sensitivity for the 30-day mortality. In addition, both CURB-65 and PSI possess the deficiency in the predictive specificity. For instance, many young patients were incorrectly categorized as low risk. More recently, SMART-COP score (Systolic blood pressure, Multilobar infiltrates, Albumin, Respiratory rate, Tachycardia, Confusion, Oxygen and pH) was derivated in Australia. SMART-COP emphasizes predicting the need for ventilatory/vasopressor support. It is still complicated to calculate multiple points for different variables and age-adjusted cut-off 7, and a further score (A-DROP: Age, Dehydration, Respiratory failure, Orientation disturbance, Systolic blood pressure) was developed in Japan^8^. All of the scores can help determine whether a patient needs to be hospitalized or even admitted to the ICU^9^.

Clearly, a simpler, but more reliable score system is needed. In this study, we evaluated multiple risk factors contributing to the 30-day mortality in hospitalized pneumonia patients coming from the community. Then we developed a simpler and more effective scoring system by expanding CURB-65, to evaluate its efficiency compared to currently available scores for severity assessment.

## Materials and Methods

### Study population

We retrospectively analyzed consecutive patients with diagnosis of CAP between January 2010 and December 2013 hospitalized at Second Affiliated Hospital, Zhejiang University School of Medicine. The definition of CAP/HCAP for this study followed the ATS and the IDSA guidelines^10^,^11^. Patients were excluded if they had HIV infection or if had been in hospital within the previous 7 days^3^. Comorbidities were documented, defined as presence of one or more of the following diseases: congestive heart failure, chronic obstructive pulmonary diseases (COPD), chronic renal diseases, chronic liver diseases, cerebrovascular diseases, malignancy (solid tumor or hematological malignancy), or diabetes mellitus^12^. The Ethics Committee of the involved hospitals approved this study.

### Clinical data

We collected all the data from each subject, including demographic factors, co-morbidity conditions, physical examination and laboratory/radiologic findings. The laboratory findings were analyzed within 24 h after admission.

### Definition of expanded-CURB-65, PSI, CURB-65, SMART-COP, and A-DROP

Severity of pneumonia was assessed using the CURB-65 score^6^, PSI score^5^, SMART-COP score^7^, A-DROP score^8^, and expanded-CURB-65 (CURB-65, lactate dehydrogenase, platelet, and albumin) we proposed, respectively.

### External validation

The new score obtained was validated on an external prospective cohort of adult patients with pneumonia hospitalized in a 1200 bed teaching hospital (Policlinico Umberto I-Rome) from Italy. Study methods were previously reported^13^,^14^. Briefly, we prospectively collected data of all episodes of pneumonia during the period between January 2013 and March 2014. All patients were followed-up to discharge or death.

### Statistical analysis

Chi-square test and Fisher’s exact test were used to determine the association between qualitative/categorical variables. Continuous variables were expressed as mean ± standard deviation (SD) and compared between groups using one-way analysis of variance. Correlations between two continuous variables were assessed with the Pearson correlation. Distribution of the analyzed continuous variables for normality was tested with the Kolmogorov-Smirnov test. Multiple logistic regression analysis was used to determine independent risk factors for mortality. The odds ratio (ORs) and corresponding 95% confidence intervals (CIs) for each variable were calculated. We evaluated discrimination using receiver operating characteristic curves (ROC) and compared the areas under the ROC curves (AUCs) for the different scores, adjusting the probability using the Sidak method. The calibration of the model was evaluated by the godness-of-fit Hosmer-Lemeshow χ^2^ statistic. All tests were two-tailed, and a *P* value < 0.05 was considered significant. Computations were carried out with SPSS 20.0 for Windows (R version 3.0.2) and STATA v.12.

## Results

### Patient characteristics

A total of 1879 hospitalized patients with CAP were evaluated in this retrospective study, and 1640 of them were finally eligible for analysis. The general characteristics of these patients are shown in Table 1. A mean age was 64 ± 19 years and 59.6% of patients were male. Overall, 37.6% of patients were accompanied by one or more coexisting illnesses, including congestive heart failure (10%), COPD (8.3%), chronic renal diseases (5.1%), chronic liver diseases (2.5%), cerebrovascular diseases (6.6%), malignancy (4.6%) and diabetes mellitus (7.9%). The median length of stay (LOS) was 10 (IQR 7–15) and the 30-day mortality was 8.48%.

### Factors related to the mortality and LOS

Thirteen potential predictive variables including all the components of CURB-65 score were selected and assessed using X^2^ test and uni- and multivariate logistic regression, respectively (see Table 2). We found that elevated serum LDH level (>230 u/L), thrombocytopenia (platelet count <10^5^/mL) and hypoalbuminemia (albumin level <3.5 g/dL) were independent risk factors for death at multivariate analysis: the Table 3 describes the prevalence of these risk factors in different risk classes of the CURB-65 and PSI scores.

### The new score system proposed for evaluating pneumonia severity

We created a new score system, which expands CURB-65 and consists of 8 parameters, named **expanded-CURB-65** including age ≥65 years, **L**DH > 230 u/L, **A**lbumin <3.5 g/dL, **P**latelet count <100 × 10^9^/L, **C**onfusion, **U**rea > 7 mmol/L, **R**espiratory rate ≥30 /min, low systolic (<90 mmHg) or diastolic (≤60 mmHg) **B**lood pressure. The expanded-CURB-65 score was categorized into three classes as follows: 0–2 as low risk, 3–4 intermediate risk, and 5–8 high risk. Accordingly, patients with one of three tiers of scores should be treated either as outpatient, or inpatients in hospital ward or ICU, respectively.

The performance in predicting the 30-day mortality and LOS by all the scoring systems is shown in Table 4, respectively. Expanded-CURB-65 scores were also positively associated with median LOS: score 0–2 (low risk) with 9 days (IQR 7–13), score 3–4 (intermediate risk) with 12 days (IQR 8–18), and score 5–8 (high risk) with 14 (IQR 9–22) (Table 4).

**Figure 1:**
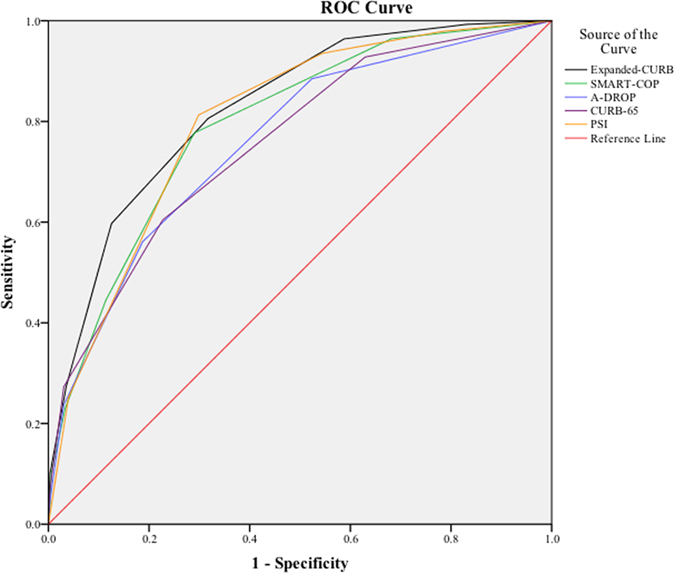
ROC curves for five scoring systems in the main cohort of CAP patients.

Finally, as described in Fig. 1, we analyzed the discrimination power of all score systems for predicting the 30-day mortality using ROC curves. Interestingly, the overall sensitivity and specificity of expanded-CURB-65 were superior (AUC = 0.826, 95% CI, 0.807–0.844) to other score systems, of which the AUCs were 0.801 (95% CI, 0.781–0.820), 0.756 (95% CI, 0.735–0.777), 0.793 (95% CI, 0.773–0.813), and 0.759 (95% CI, 0.737–0.779) for PSI, CURB-65, SMART-COP and A-DROP, respectively. The expanded-CURB-65, CURB-65, A-DROP, and PSI were validated in CAP (P = 0.336, 0.157, 0.178, 0.576, respectively) by the Hosmer-Lemeshow test after Bonferroni correction. SMART-COP was not validated among CAP patients after Bonferroni correction (P = 0.008).

### Validation of the new score system proposed

**Figure 2:**
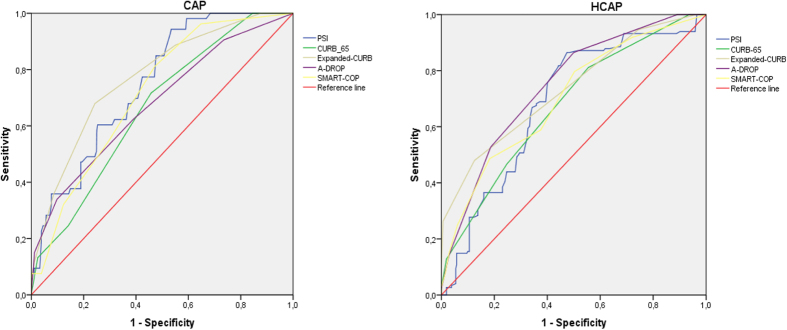
ROC curves for five scoring systems in CAP and HACP patients in the validation cohort.

The performance of the expanded-CURB was evaluated in a validation cohort of 1164 patients with pneumonia; out of these 39.5% fulfilled HCAP definition. Compared to the main cohort, these patients were older (mean age 75, P < 0.001) and had a slightly higher 30-day mortality (17.3%, P < 0.001). The performance of this expanded CURB-65 score was confirmed well in all patients of the validation cohort (AUC 0.78, 95% CI, 0.746–0.814). Among CAP patients, AUCs for prediction of 30-day mortality were 0.772 (95% CI, 0.710–0.834), 0.748 (95% CI, 0.691–0.806), 0.663 (95% CI, 0.599–0.728), 0.716 (95% CI, 0.655–0.776), and 0.679 (95% CI, 0.602–0.755) for the expanded–CURB–65, PSI, CURB-65, SMART-COP and A-DROP, respectively. For HCAP patients, AUCs were 0.736 (95% CI, 0.687–0.784), 0.686 (95% CI, 0.635–0.737), 0.675 (95% CI, 0.626–0.724), 0.700 (95% CI, 0.650–0.751), and 0.747 (95% CI, 0.703–0.791), respectively. Once again, there were no significant differences in term of AUCs between the main cohort and the validation cohort. Using the Hosmer-Lemeshow test, we found that expanded-CURB-65 is validated on CAP (P = 1.000 after Bonferroni correction), but not on HCAP patients (P = 0.0175 after Bonferroni correction). In addition, we had satisfactory calibration of CURB-65, SMART-COP and A-DROP (P = 0.411, 0.225, 0.695, respectively) among CAP patients, but not of PSI (P = 0.0035). Among HCAP patients, we had good calibration of CURB-65 and A-DROP (P = 0.740 and 1.000, respectively) but not of PSI (P < 0.001) and SMART-COP (P = 0.0095). Figure 2 summarizes ROC curves of all score systems in both CAP and HCAP groups, respectively.

## Discussion

The purpose of our study was to establish and evaluate a new simpler and more efficient scoring system for assessing severity of CAP. We first identified 8 independent risk factors closely related to the mortality of CAP patients. Then, a new severity score termed expanded-CURB-65, consisting of these 8 variables, was tested in this cohort. Our results showed that this new system was relatively simpler, but more efficient than those early established, and its efficacy was confirmed in an external validation cohort.

Three key factors seem to improve performance of CURB-65 score: LDH levels, thrombocytopenia, and hypoalbuminemia. LDH is a cytoplasmatic enzyme expressed in nearly all types of cells of the body. It is released into blood when cell experiences injury or death caused by ischaemia, excess heat or cold, starvation, dehydration, injury, bacterial toxins, drugs and chemical poisonings^15^,^16^,^17^. Because of higher concentration expressed in various organs/tissues, the leakage of LDH from even a small scale of injured tissue can result in a significantly elevated serum level^15^. LDH has been used as an indicator of cellular injury induced by various etiologies. Previous studies have demonstrated that the elevated LDH in serum, especially in bronchoalveolar lavage (BAL) and pleural fluid can help determine the extent of lung tissue damage and inflammation such as pulmonary embolism*, P. carinii* pneumonia, tuberculosis, bacterial pneumonia, influenza A^18^,^19^,^20^,^21^,^22^,^23^. Ewig reported that the increased serum LDH values were associated with increased mortality in 92 CAP patients^24^. The higher serum LDH level indicates the more severe complications and the worse prognosis.

Thrombocytopenia and hypoalbuminemia also serve as markers indicating that patients need ICU admission and may result in a higher mortality in hospitalized CAP patients^25^,^26^,^27^,^28^. Platelets, hemostasis, and wound healing are all involved in inflammatory lung diseases^29^,^30^, and appearance of thrombocytopenia, which results in coagulation derangements, is associated with dismal prognosis in CAP patients and it is one of the minor criteria for severe CAP by IDSA/ATS^10^. Hypoalbuminemia, which can be caused by malnutrition, liver cirrhosis, or infection process, contributes to an increased mortality in hospitalized patients^31^, and several studies have identified a close correlation between serum albumin concentration and mortality in CAP patients^6^,^7^,^28^,^32^,^33^. Therefore, expanding CURB-65 through including LDH, thrombocytopenia and hypoalbuminemia levels can increase the efficiency of predicting the severity in CAP patients. Recently, the studies suggested that either pre-existing diabetes or acute hyperglycemia without pre-existing diabetes was associated with longer LOS and higher mortality in CAP patients^34^,^35^. However, our data did not show a positive correlation between hyperglycemia and the 30-day mortality.

During last decades, several quantitative score systems, including PSI, CURB-65, SMART-COP and A-DROP, have been developed to assess pneumonia severity. PSI, which draws the score from 20 variables, is accurate in predicting the 30-day mortality, but its complexity limits clinical application. Contrarily, CURB-65 features simplicity. However, the patient’s age and complications in both PSI and CURB65 carry heavier weight, underestimating the potential severity in young patients and falsely referring the elderly CAP patients as severe^36^. Moreover, both PSI and CURB-65 scores did not exhibit good performance in stratifying risk of death among patients with community-onset pneumonia but fulfilling HCAP definition^37^. In addition, neither PSI nor CURB-65 was designed to identify patients who need to be referred to the ICU, and SMART-COP was aimed to compensate this function^7^. The SMART-COP, which is processed by logistic regression analysis, provides superior accuracy for prediction of the need for intensive respiratory or vosopressor support, but it is a still complicated process to calculate multiple points for different variables and age-adjusted cut-off. For CAP patients in Asia, A-DROP is a simple modified version of the CURB-65 and it is recommended by Japan Respiratory Society (JRS), but the ROC curve for A-DROP is similar to CURB-65^12^.

Compared to CURB-65 and other assessment tools, the expanded-CURB-65 score, which extends independent risk factors to 8 variables in assessing CAP severity, significantly improves identifying high-risk patients, through decreasing the relative weight of age and blood pressure, and eliminating the use of imaging and comorbid illnesses in the calculation. The only gap remains among HCAP patients, since data from validation cohort did not confirm a good performance of the score in this subgroup of patients. HCAP comprises a heterogeneous population of patients with more comorbidities, recent healthcare contacts and increased risk of multidrug resistance pathogens. Probably this category of patients is quite different to that considered as “classical” CAP, and some authors have proposed to reconsider this epidemiological entity^38^. The findings of our study confirm the need of further characterization of the subgroup of patients fulfilling HCAP definition, to improve the initial assessment and management of this category.

There are a few limitations in this study. First, this is a retrospective single center study and no outpatients were included. Second, although the blood samples were collected as soon as patients were admitted, there were variations in the timing of collecting. In addition, we didn’t include the ICU admission as a risk factor because a portion of patients died before reaching the ICU due to the shortage of ICU resources and financial support in developing countries. However, our data were validated on a prospective cohort of patients with pneumonia observed in a geographic area completely different in term of demographic features, predisposing factors, and hospital resources. Thus we believe that it is the major strength of our study.

In conclusion, our expanded CURB-65 is a relatively simpler and more effective marker in assessing the severity of hospitalized patients with CAP. Despite the encouraging results, further validation is warranted in future multicenter large prospective studies.

## Additional Information

**How to cite this article**: Liu, J.-l. *et al*. Expanded CURB-65: a new score system predicts severity of community-acquired pneumonia with superior efficiency. *Sci. Rep.* **6**, 22911; doi: 10.1038/srep22911 (2016).

## Figures and Tables

**Table 1:** Baseline characteristics of CAP patients.

Demographic data	
Age, mean (±SD), y	64 ± 19
Age ≥ 65 years, N (%)	881 (53.7)
Male, N (%)	977 (59.6)
Comorbidities, N (%)	616 (37.6)
Congestive heart failure	164 (10.0)
COPD	136 (8.3)
Chronic renal diseases	83 (5.1)
Chronic liver diseases	41 (2.5)
Cerebrovascular diseases	109 (6.6)
Malignancy	75 (4.6)
Diabetes mellitus	130 (7.9)
Physical examination findings N (%)	
Confusion	85 (5.2)
Respiratory rate ≥ 30 /min	39 (2.4)
Heart rate ≥125 /min	36 (2.2)
Blood pressure (systolic < 90 mmHg or diastolic ≤ 60 mmHg)	127 (7.7)
LOS (interquartile range)	10 (7–15)

**Table 2:** Univariate and multivariate analyses of features associated with 30-day mortality in CAP patients.

**Risk factor**	**Univariate analysis**			Multivariate logistic regressionanalysis			
	**OR**	**95**% CI	**p**	**β**	**OR**	**95**% CI	**P**
Age ≥ 65 years	2.89	1.93–4.30	<0.01	0.67	1.95	1.25–3.03	<0.01
BUN > 7 mmol/L	4.82	3.39–6.92	<0.01	0.98	2.68	1.79–4.01	<0.01
Confusion	7.24	4.45–11.76	<0.01	1.48	4.39	2.52–7.65	<0.01
Respiratory rate ≥ 30 /min	6.61	3.35–13.04	<0.01	1.34	3.82	1.68–8.74	<0.01
Blood pressure(SBP < 90 mmHg orDBP ≤ 60 mmHg)	3.01	1.87–4.85	<0.01	0.63	1.88	1.06–3.32	0.03
Pulse ≥ 125 beats/min	3.78	1.74–8.21	<0.01	0.91	2.49	0.95–6.55	0.06
Serum LDH level > 230U/L	3.65	2.52–5.29	<0.01	0.73	2.07	1.37–3.11	<0.01
Albumin level <3.5 g/dL	3.59	2.36–5.47	<0.01	0.72	2.06	1.29–3.28	<0.01
Platelet count <10^5^/mL	4.94	3.42–7.13	<0.01	1.05	1.05	1.88–4.32	<0.01
Glucose level ≥ 11.1 mmol/L	2.38	1.48–3.84	<0.01	0.13	0.13	0.65–2.02	0.65
WBC count <4 or >10 × 10^9^/L	1.49	1.05–2.11	<0.01	0.12	0.12	0.76–1.70	0.55
C-reactive protein level >150 mg/L	2.11	1.46–3.06	<0.01	0.21	0.21	0.80–1.90	0.06

**Table 3:** The prevalence of hypoalbuminemia, thrombocytopenia and high serum LDH in different risk classes of the CURB-65 and PSI scores in CAP patients.

**Subgroup**	Patients, N(%)	SerumLDH > 230 u/L,N (%)	Hypoalbuminemia,N (%)	Thrombocytopenia,N (%)
PSI class				
I–II	693 (42.26)	219 (31.60)	276 (39.83)	57 (8.23)
III	386 (23.54)	153 (39.64)	227 (58.81)	59 (15.28)
IV–V	561 (34.21)	268 (47.77)	378 (67.38)	144 (25.67)
CURB-65 class				
0–1	1215 (74.09)	417 (34.32)	599 (49.30)	147 (12.10)
2	341 (20.79)	172 (50.44)	223 (65.40)	87 (25.51)
3–5	84 (5.12)	51 (60.71)	59 (70.24)	26 (30.95)

**Table 4:** Outcome of subgroups for PSI, CURB-65, SMART-COP, A-DROP and expanded CURB-65 scores in CAP.

**Subgroup**	**Patients, N (%)**	LOS, Median(IQR)	Mortality, N(%)
PSI class			
I–II	693 (42.26)	9 (6–13)	9 (1.30)
III	386 (23.54)	12 (7–15)	17 (4.40)
IV–V	561 (34.21)	15 (8–18)	113 (20.14)
CURB-65 class			
0–1	1215 (74.09)	9 (7–14)	55 (4.53)
2	341 (20.79)	12 (8–18)	46 (13.49)
3–5	84 (5.12)	12 (7–17.75)	38 (45.24)
SMART-COP class			
0–2	1406 (85.73)	10 (7–14)	77 (5.48)
3–4	211 (12.87)	12 (8–19)	48 (22.75)
5–8	23 (1.40)	10 (5–16)	14 (60.87)
A-DROP class			
0–1	1281 (78.11)	9 (7–14)	61 (4.76)
2	280 (17.07)	11 (8–17.75)	45 (16.07)
3–5	79 (4.82)	10 (7–17)	33 (41.77)
Expanded CURB-65 class			
0–2	1052 (64.15)	9 (7–13)	27 (2.57)
3–4	497 (30.30)	12 (8–18)	74 (14.89)
5–8	91 (5.55)	14 (9–22)	38 (41.76)
