# Corrigendum: Expanded CURB-65: a new score system predicts severity of community-acquired pneumonia with superior efficiency

**DOI:** 10.1038/srep47005

**Published:** 2018-08-09

**Authors:** Jin-liang Liu, Feng Xu, Hui Zhou, Xue-jie Wu, Ling-xian Shi, Rui-qing Lu, Alessio Farcomeni, Mario Venditti, Ying-li Zhao, Shu-ya Luo, Xiao-jun Dong, Marco Falcone

Scientific Reports 6: Article number: 2291110.1038/srep22911; published online: 03 18 2016; updated: 08 09 2018

This Article contains typographical errors in Table 2 in the Multivariate logistic regression analysis section. The OR values for Platelet count, Glucose level, WBC count and C-reactive protein level are duplicated from their partial regression coefficients (β).

The correct values should read 2.85, 1.14, 1.13, and 1.23 respectively.

